# In vitro and in vivo phosphorylation of the Ca_v_2.3 voltage-gated R-type calcium channel

**DOI:** 10.1080/19336950.2018.1516984

**Published:** 2018-09-29

**Authors:** T. Schneider, S. Alpdogan, J. Hescheler, F. Neumaier

**Affiliations:** Center of Physiology and Pathophysiology, Institute of Neurophysiology, Cologne, Germany

**Keywords:** Covalent modification, exon skipping, facilitation, protein kinase A, protein kinase C, splice variants

## Abstract

During the recording of whole cell currents from stably transfected HEK-293 cells, the decline of currents carried by the recombinant human Cav2.3+β3 channel subunits is related to adenosine triphosphate (ATP) depletion after rupture of the cells. It reduces the number of functional channels and leads to a progressive shift of voltage-dependent gating to more negative potentials (Neumaier F., et al., 2018). Both effects can be counteracted by hydrolysable ATP, whose protective action is almost completely prevented by inhibition of serine/threonine but not tyrosine or lipid kinases. These findings indicate that ATP promotes phosphorylation of either the channel or an associated protein, whereas dephosphorylation during cell dialysis results in run-down. Protein phosphorylation is required for Ca_v_2.3 channel function and could directly influence the normal features of current carried by these channels. Therefore, results from in vitro and in vivo phosphorylation of Ca_v_2.3 are summarized to come closer to a functional analysis of structural variations in Ca_v_2.3 splice variants.

## Introduction

Covalent modification of ion channels and other proteins is a common event during cellular growth, metabolic regulation and neuronal signalling. Many cell signalling pathways act on voltage-gated calcium channels to fine tune their activities and to control their cell surface expression. They are often modulated via G protein-coupled receptors, which control downstream events by posttranslational modification including phosphorylation of the pore-forming Ca_v_α_1_-subunit or interacting auxiliary proteins [1].

The exact molecular mechanisms of these biochemical changes are only partially known for individual calcium channel subtypes. Best investigated are certain members of the L-type (Ca_v_1) subfamily, which comprises the skeletal muscle Ca_v_1.1 subunit and three additional homologous subunits (Ca_v_1.2, Ca_v_1.3 and Ca_v_1.4). They are expressed in various tissues including cardiac, smooth muscle and neuronal regions (for details see [2,3]). It was the sympathetic regulation of cardiac activity [4], which was investigated first and most intensive to understand how Ca_v_1.2 calcium channels may modulate heart beat frequency and power during rest and working activity (for details [5]).

Members of the second calcium channel subfamily (Ca_v_2) are mainly expressed in the neuronal system [6] and were early analyzed as *in vitro* phosphorylation targets of protein kinase A [7]. Ca_v_2.3 containing voltage-gated calcium channels are known since 1993, when the first clone was found in *Discopyge ommata* [8]. During the same time a mammalian counterpart was identified by electrophysiological measurements [9,10]. Initially, it was proposed that the Ca_v_2.3-gene (cacna1e) may encode a structurally unknown T-type calcium channel [11]. However, depending on the recording conditions in recombinant systems, the human counterpart proved to be a mid-voltage gated calcium channel [12,13].

Members of the third calcium channel subfamily (Ca_v_3) show unique gating properties, which make them important for neuronal spontaneous firing, for pacemaker activities, for rebound burst firing and additional oscillating processes [14]. They contribute to hyperexcitability disorders [15,16] such as epilepsy [17] and their blockade prevents tonic-clonic seizures [18]. At physiological temperatures (30 – 37°C), activation of PKA as well as PKC (but not PKG) result in a potent increase of currents mediated by the members of this subfamily (Ca_v_3.1, Ca_v_3.2 and Ca_v_3.3) [18]. So far, the Ca_v_3.2 T-type Ca^2+^ channel is the only voltage-gated Ca^2+^-channel, which was immunopurified from rat brain to identify *in vivo* phosphorylation sites by high-resolution mass spectroscopy [19]. Interestingly, a hot spot locus of phosphorylation sites in the I-II loop was found to be critical for the channels regulation. Even more important, within the 34 sites found *in vivo*, a total of 26 sites was never described among *in vivo* phosphosites and 4 sites were not even predicted to be phosphorylated by widely used computer algorithms, underscoring the limitations of algorithm-based predictions [19].

## Physiological and pathophysiological roles of Ca_v_2.3/R-type voltage-gated Ca^2+^ channels

Using subtype-specific antibodies, the regional and subcellular localization of Ca_v_2.3 was characterized in mice and rats at both light and electron microscopic levels [20]. Ca_v_2.3 immunogold particles were found to be predominantly presynaptic in the interpeduncular nucleus, but postsynaptic in other brain regions. Serial section analysis of electron microscopic images from the hippocampal CA1 region revealed a higher density of immunogold particles in the dendritic shaft plasma membrane compared with the pyramidal cell somata [20].

Ca_v_2.3 channel expression appears to be required for long term potentiation (LTP) induced by short brief tetani [21], as presynaptic LTP and posttetanic potentiation (PTP) are impaired in Ca_v_2.3-deficient mice as well as in control mice which were pharmacologically antagonized [21,22].

Additional evidence that Ca_v_2.3 channels contribute to neurotransmission arises from expression studies in neurons. Exogenous Ca_v_2.3 channels expressed by cDNA injection in cultured superior cervical ganglion neurons mediate cholinergic synaptic transmission [23]. This supports earlier results from Wu et al. (1998) who demonstrated that calcium influx through Ca_v_2.3 channels is able to evoke transmitter release in the calyx of Held from young mice large enough to initiate an action potential in the postsynaptic neuron. However, transmitter release mediated by Ca_v_2.3 channels is less efficient than transmitter release by Ca_v_2.1 (P/Q-) and Ca_v_2.2 (N-type) Ca^2+^ currents. The exact mechanisms underlying these differences are not fully clear [24].

The coupling to synaptic transmission varies within the subfamily of Ca_v_2 channels over different frequency ranges with consequences for the frequency tuning of both synaptic dynamics and presynaptic neuromodulation [25]. Relative to Ca_v_2.1, Ca_v_2.2 had a disproportionately reduced contribution to synaptic transmission at frequencies >20 Hz, while Ca_v_2.3 had a disproportionately increased contribution to synaptic transmission at frequencies >1 Hz. These activity-dependent effects of different Ca_v_2 family members shape the filtering characteristics of GABAB receptor-mediated presynaptic inhibition [25].

Mice lacking the Ca_v_2.3 channel showed altered pain responses. Obviously, the Ca_v_2.3 channel controls pain behavior by both spinal and supraspinal mechanisms [26]. In HEK-293 cells expressing members of the Ca_v_2 subfamily, the mechanism of Ca^2+^ channel modulation by opioid receptor (OR) activation was analyzed and the different types of ORs were cotransfected with Ca_v_2.3. Selective agonists of µ-, δ-, and κ-ORs inhibited I_Ba_ through Ca_v_2.3 channels by 35%. Ca_v_2.2 channels were inhibited more potently [27].

Within several unexpected tissues the ablation of Ca_v_2.3 channels disturbed the regularity of rhythmic processes like heart beat [28–30], peptide hormone secretion [31–33] and sensory signal transduction [34,35]. Depending on the mouse model used for ablation of Ca_v_2.3, opposite results have been reported with regard to the role of Ca_v_2.3 channels during sleep [36,37].

The general ablation of Ca_v_2.3 channels alters seizure susceptibility after treatment with kainate in mice, so that hippocampal seizure resistance is increased [38] and neuronal excitotoxicity reduced [39]. Thus, the molecular understanding of the Ca_v_2.3 channel gating and its modulation is needed to adopt future therapies for cardiac, endocrinal and neuronal diseases.

## Pharmacological properties of Ca_v_2.3

When the primary sequence for the ray Ca_v_2.3 was deduced and its cRNA was functionally expressed in *Xenopus* oocytes, it was finally described as a so called “pharmacoresistant” (R-type) voltage-gated Ca^2+^ channel. In cerebellar granule cells the counterpart for the ray version was identified and its kinetic and pharmacological properties differed from the known Ca^2+^ subtypes [9,10]. In 1998 the peptide toxin SNX-482 was identified in the tarantula *Hysterogrates gigas*, which blocked in rat neurophypophyseal nerve terminals at low nanomolar concentrations the R-type Ca^2+^ currents, but not in several types of rat central neurons [40]. Later on, it was found that SNX-482 dramatically reduces the A-type K^+^ current in acutely dissociated dopamine neurons from the mouse substantia nigra pars compacta with an IC_50_ of less than 3 nM [41]. Therefore, a highly selective antagonist of Ca_v_2.3/R-type currents is not available today but needed urgently.

Ca_v_2.3 channels are very sensitive towards divalent metal cations, similar as some T-type channels [42,43]. Further, testing the convulsive drug kainate, which is routinely used for experimentally induced epilepsy, led us to the conclusion that the sensitivity of Ca_v_2.3 channels towards divalent bioavailable metal cations must be investigated more in detail to understand physiological imbalances of Zn^2+^ and Cu^2+^ concentrations [44] in neuronal and other tissues, which may explain changes in excitability causing neurological and other diseases.

As an important side remark, recombinant Ca_v_2.3 Ca^2+^ channels represent low affinity dihydropyridine receptors for nicardipine in COS-7 cells [45] and for racemic isradipine in HEK-293 cells [28]. Therefore, it may not be excluded that the therapeutical use of high local dihydropyridine concentrations after subarachnoidal hemorhage (SAH) do target the Ca_v_2.3/R-type channel, which increases its expression after SAH [46–48].

### Early investigations of Ca_v_2.3 phosphorylation

Early after the detection of the pharmacoresistant Ca^2+^ channel types and based on the published deduced primary sequences, channel-specific antibodies were raised and used for *in vivo* characterization of Ca_v_2.3 expression in the rat brain [49]. The authors determined in partially purified preparations the phosphorylation of the anti-Ca_v_2.3 immunopositive protein(s) by 4 different kinases, comprising cAMP-dependent protein kinase (PKA), cGMP-dependent protein kinase (PKG), protein kinase C (PKC) and Ca^2+^/calmodulin-dependent protein kinase II (CaMKII). Immunoblotting identified a polypeptide of 245 to 255 kDa, which was not a high affinity receptor for classical Ca^2+^ channel blockers [49].

## Prediction of putative phosphorylation sites increases over time

As soon as the deduced primary sequences of newly cloned voltage-gated Ca^2+^ channels became identified, software packages (Genetics Computer Group, 1991) could be used to predict putative phosphorylation sites within the deduced sequences. For example, more than a dozen different sites for PKA are predicted to be present in the human Ca_v_2.3d variant, 3 of them located in the I-II loop (close to a putative EF-hand), 4 of them in the II-III loop, one of them in the distal and a cluster of 6 in the proximal C-terminus [13]. Meanwhile, about 10 different online predictors are available as software and the number of potential sites for phosphorylation by various protein kinases has increased dramatically. According to the information on their website (http://gps.biocuckoo.org/index.php), the newly released Group-based Prediction System (GPS 3.0) covers a novel peptide selection and more than 6000 phosphorylation sites for training, allowing for the prediction of kinase-specific phosphorylation sites for 464 human protein kinases in hierarchy.

Using the human primary Ca_v_2.3 sequence of the fetal brain (Ca_v_2.3d, L27745.2) at the highest prediction threshold, the software predicts a total of 95 consensus sites for the cAMP-dependent protein kinase. Thus, the number of predicted sites is large and cannot be tested easily. But *in vivo* analysis as done recently for the Ca_v_3.2/T-type channel [19] could be performed as a more reliable approach.

## Functional differences of recombinant Ca_v_2.3 splice variants modulated by phorbol ester stimulation and protein kinase c inhibition

The subfamily Ca_v_2 of voltage-gated calcium channels had been investigated more systematically for its function as targets for PKC-dependent modulation in combination with a molecular crosstalk to other second messenger pathways [50]. For the tested recombinant channel types in *Xenopus* oocytes, only Ca_v_2.2 and Ca_v_2.3 but not Ca_v_2.1 were sensitive towards PKC activation by PMA (phorbol 12-myristate 13 acetate) [51]. During these investigations, the cytosolic loop between domain I and II was identified as an important integration center between PKC-mediated activation and G protein dependent modulation of the Ca_v_2.2/N-type Ca^2+^ channels [52].

Structure – function relation of the PKC-mediated Ca_v_2.3 activation was investigated in more detail in HEK-293 cells when comparing the transient facilitation of different Ca_v_2.3 splice variants by Ca^2+^-influx. With Ba^2+^ as charge carrier, the tested biophysical properties were similar. In Ca^2+^, the inactivation time course was slower and the recovery from short-term inactivation was faster only in splice variants containing a 19-amino-acid-long arginine-rich insertion, which is typical for subset of neuronal Ca_v_2.3 calcium channel subunits [53–55].

The mechanism underlying the Ca^2+^ dependent increase of channel activity was investigated by introduction of the II-III loop of Ca_v_2.1 channels, which abolished the transient Ca^2+^ mediated stimulation as well as the phorbol ester (PMA or PDBu) sensitivity of the resulting construct [56]. Also the 19 amino acids long arginine-rich insertion (encoded by exon 19) was needed in the recombinant system for PKC-mediated stimulation of Ca_v_2.3, leading to the conclusion that only the splice variants Ca_v_2.3c and Ca_v_2.3d (both containing exon 19 encoded sequences) are subject to Ca^2+^ and phorbolester mediated stimulation. Interestingly, the arginine-rich segment contains no serine or threonine residues, as would be expected if PKC-mediated stimulation involves phosphorylation of this region. Instead, it could harbor a PKC binding site or other determinants for the association of Ca_v_2.3 channels with PKC, which may be required for phosphorylation of other sites in the Ca_v_2.3 channel protein. This interpretation is partly supported by the observation that the FLAG-tagged II-III loop of Ca_v_2.3 augments the autophosphorylation of exogenously added PKCα [57].

During the years several serine/threonine sites in Ca_v_2.3 were identified, which mediate stimulatory and inhibitory effects of protein kinase C isozymes (for an overview see [58]). Some sites reside in the I-II linker, where G-protein binding was identified close to the predicted phosphorylation sites for the other two members of the Ca_v_2 subfamily [59–61]. Interestingly, the effects on Ca_v_2.3 are dependent on the PKC-isozyme used for activation, and PKC mediates stimulatory and inhibitory effects on Ca_v_2.3-mediated currents [58,62].

## Quantitative phospho-proteomic studies get closer to the *in vivo* situation

Compared to other posttranslational modifications, protein phosphorylation causes one of the most widespread covalent structural changes, which involves transfer of a negatively charged phosphate group to certain amino acid side chains of newly synthetized proteins [63]. Apart from the formation of acid stable phosphomonoesters of serine, threonine and tyrosine, other less stable covalent modifications have been described, such as low stability phosphohistidine residues, which are problematic to analyze by conventional proteomic methods [64,65]. A quantitative phosphoproteomic analysis is highly useful for identifying and characterizing changes in protein function and for mapping associated regulatory pathways. Mass spectrometric analysis of stable isotope labelled samples has progressed and meanwhile the enrichment of phosphopeptides has improved to a level that allows for identification of less stable and low abundant phospopeptides. The new techniques pay attention to the fact that *in vivo* phosphorylation of a target protein is often a transient event [66–68] and typically occurs at low stoichiometry [65]. Also, phosphoproteomic analyzes initially restricted to single organs of the mouse [69] have been extended to large scale human interactome analyzes [70], which provide insights into hundreds of poorly characterized proteins and interconnecting signal transduction pathways.

Interestingly, patterns of Ca^2+^ channel phosphorylation observed in phosphoproteomic analyzes reveal a clustering of phosphorylation sites at two cytoplasmic regions of the Ca_v_α_1_-subunits, which comprise the II-III loop and the carboxy terminus [71]. For the Ca_v_2.3 α1-subunit, most identified phosphorylation sites are located within the II-III loop (Table 1).

**Table 1.** **Phosphorylation sites identified in the mouse brain Ca_v_2.3 α_1_-subunit (Q61290)** [71]. The GenBank entry Q61290 mentions only part of the sites, which were originally listed as *in vivo* identified phosphopeptides (labelled in bold). In addition, this so called large scale analysis identified residue **Ser-816** in the II-III loop as an additional phosphorylation site.

Location within the Ca_v_2.3 α_1_-subunit:				
N-terminus	I-II loop	II-III loop	III-IV loop	C-terminus
**Ser-15** **Ser-20** Thr-29	**Ser-428** **Thr-441**	Ser-737 Ser-740 **Ser-746** Ser-793 Ser-794 **Ser-856** Thr-865 Ser-866 Ser-873 Ser-876 Ser-1051 Ser-1056 Thr-1094	– none -	**Ser-2054** Thr-2067 Ser-2073

For the vertebrate Ca_v_2.3 at least 3 major splice variants (Ca_v_2.3c, Ca_v_2.3d, and Ca_v_2.3e) have been identified by cloning and RT-PCR. They have been characterized electrophysiologically showing significant differences for the fast component of the time constants for the recovery from short-term inactivation, when lacking the arginine-rich insert within the II-III loop [53].

**Figure 1.**
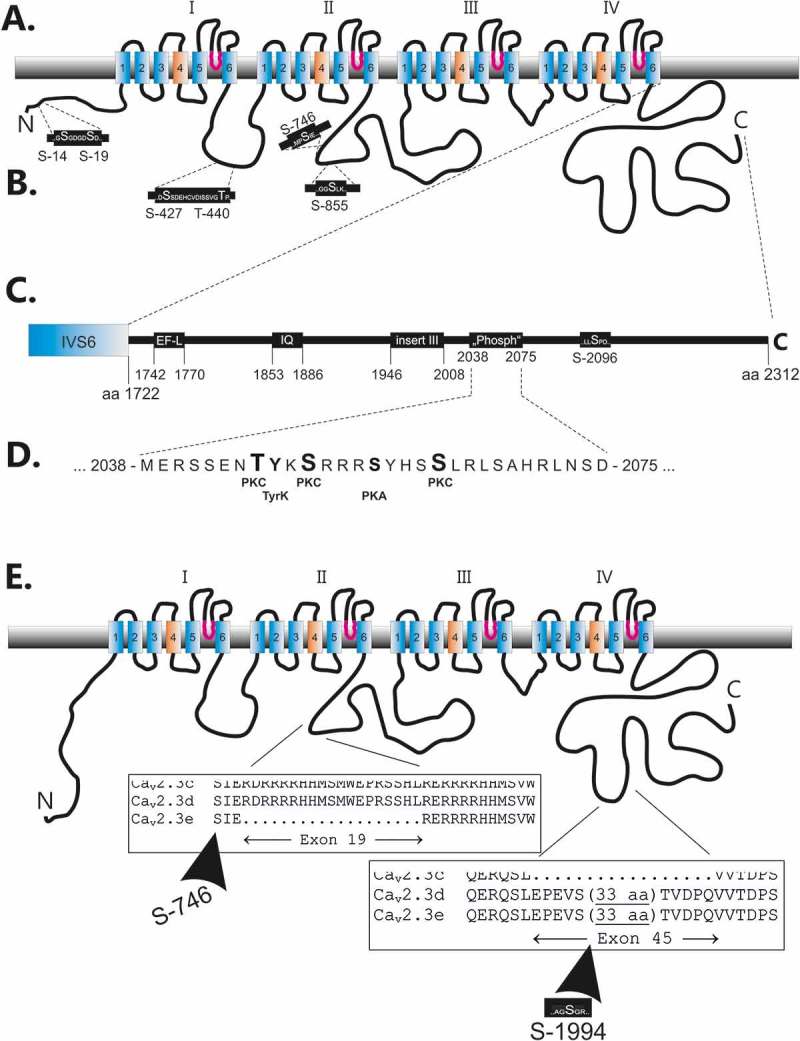
Location of predicted phosphorylation sites within human Ca_v_2.3d. (a). Cartoon of the deduced transmembrane organization of the human Ca_v_2.3d subunit. (b). Predicted phosphorylation sites within the amino terminus, the I-II loop and the II-III loop. (c). and (d). Predicted phosphorylation sites within the carboxy terminus including a cluster of putative phosphorylation sites for PKC, PKA and tyrosine kinase downstream of important functional domains (EF-like and IQ site as well as the inserted exon 45 of the splice variant d of Ca_v_2.3). (E). A detailed view of the alternate exons 19 and 45, which are identified in different Ca_v_2.3 splice variants (for details see 53) is shown. Note within exon 45 one additional PKC site, which was predicted.

One of the *in vivo* identified phosphopeptides of the II-III loop from the mouse brain (Ser-746) is located in close neighbourhood to the arginine-rich insert of the II-III loop, which is encoded by the alternative exon 19 (Figure 1(b) and 1(e)).

Another remarkable but only putative phosphorylation site, predicted to be phosphorylated by protein kinase C, is located within the alternative exon 45 (Figure 1(e)). So far, no homologous phosphopeptide was identified in mouse brain studies.

Cloned human Ca_v_2.3d voltage gated calcium channels [13], co-expressed with human β_3_ [72], are regulated by protein kinases and phosphatases in HEK-293 cells [73]. This is reflected in a spontaneous decline of current responses (run-down) and changes in channel gating in dialysed cells, which can be prevented by provision of hydrolysable ATP. Importantly, the protective action of ATP is prevented by inhibition of serine/threonine but not tyrosine or lipid kinases, suggesting that protein phosphorylation is required to prevent rapid de-phosphorylation by one or more associated protein phosphatases and thus maintain the normal function of Ca_v_2.3 channels.

It will be a future challenge to correlate the identified putative and real phosphorylation sites with observed molecular movements in the channel protein structure, in order to understand the macroscopic changes in function of the body.
